# Free Fatty Acids Differentially Downregulate Chemokines in Liver Sinusoidal Endothelial Cells: Insights into Non-Alcoholic Fatty Liver Disease

**DOI:** 10.1371/journal.pone.0159217

**Published:** 2016-07-25

**Authors:** Rachel H. McMahan, Cara E. Porsche, Michael G. Edwards, Hugo R. Rosen

**Affiliations:** 1 Department of Medicine, Division of Gastroenterology and Hepatology, University of Colorado Denver, Aurora, Colorado, United States of America; 2 Department of Medicine, Division of Pulmonary Sciences and Critical Care Medicine, University of Colorado Denver, Aurora, Colorado, United States of America; 3 Department of Microbiology and Immunology, University of Colorado Denver, Aurora, Colorado, United States of America; 4 Denver Veteran’s Affairs Medical Center, Denver, Colorado, United States of America; INRA, FRANCE

## Abstract

Non-alcoholic fatty liver disease is a prevalent problem throughout the western world. Liver sinusoidal endothelial cells (LSEC) have been shown to play important roles in liver injury and repair, but their role in the underlying pathogenetic mechanisms of non-alcoholic fatty liver disease remains undefined. Here, we evaluated the effects of steatosis on LSEC gene expression in a murine model of non-alcoholic fatty liver disease and an immortalized LSEC line. Using microarray we identified distinct gene expression profiles following exposure to free fatty acids. Gene pathway analysis showed a number of differentially expressed genes including those involved in lipid metabolism and signaling and inflammation. Interestingly, in contrast to hepatocytes, fatty acids led to decreased expression of pro-inflammatory chemokines including CCL2 (MCP-1), CXCL10 and CXCL16 in both primary and LSEC cell lines. Chemokine downregulation translated into a significant inhibition of monocyte migration and LSECs isolated from steatotic livers demonstrated a similar shift towards an anti-inflammatory phenotype. Overall, these pathways may represent a compensatory mechanism to reverse the liver damage associated with non-alcoholic fatty liver disease.

## Introduction

Non-alcoholic fatty liver disease (NAFLD) is the fastest-growing liver disease in the western world. NAFLD represents a disease spectrum that is histologically defined and can range from simple hepatic steatosis to non-alcoholic steatohepatitis (NASH). NASH includes steatosis along with liver inflammation, hepatocyte injury and often fibrosis [1]. The mechanisms that lead to the different pathological outcomes are not well defined but hepatic lipid accumulation, primarily as triacylglycerol (TAG), is a key pathogenic feature of NAFLD [2]. Hepatic TAG synthesis results in part from increased uptake of hepatic fatty acids. Circulating free fatty acids (FFA) make up the majority of FFA encountered by the liver [3] although they can also originate from de novo lipogenesis (DNL) in hepatocytes [4]. In the context of NAFLD elevated plasma FFA are observed and are at least one source for TAG synthesis in hepatocytes [5]. In addition, elevated levels of the saturated palmitic acid (PA) and the mono-unsaturated oleic acid (OA) are found in NAFLD patients and make up a majority of the FFA in TAG [3,6]. In vitro studies of the cellular and metabolic effects of FFA on hepatic cells have focused primarily on hepatocyte function with some studies showing lipotoxic effects on hepatocytes [7–9] and others suggesting that the effects vary depending on the composition of the FFA [10].

Liver sinusoidal endothelial cells (LSEC) play an important role in the regulation of the transport of macromolecules between the blood and liver parenchyma including lipids and lipoproteins. LSEC lack a basement membrane and have pores or fenestrae which allow for regulation of macromolecule transport [11]. In addition, LSEC are also important regulators of lymphocyte adhesion and migration across the sinusoidal endothelium into the parenchyma via expression of adhesion molecules and chemokines [12]. It has been demonstrated both in tissue culture and in murine hepatotoxicity fibrosis models that LSEC can produce a number of inflammatory mediators including pro-inflammaotry cytokines (TNF**α**, IL-6 and IL-1) and chemokines (CCL2 (MCP-1), CCL3 (MIP1**α**), CCL4 (Mip1**β**), CCL5 (Rantes), CXCL1 (KC), CXCL2 (MIP2**α**))[13,14]. Taken together this suggests that LSEC may participate in the inflammatory response associated with NAFLD. The aim of current study was to evaluate the effects of FFA on LSEC phenotype including cell survival, lipid metabolism and inflammatory mediators in both primary LSEC and immortalized LSEC lines. Here we show that, in contrast to hepatocytes, FFAs inhibit LPS-induced pro-inflammatory chemokine production in LSEC and inhibit inflammatory cell recruitment. This data suggests that LSEC could potentially play a protective role when the liver is presented with an overabundance of FFA as seen in NAFLD.

## Materials and Methods

### Cell Lines and Animals

The immortalized murine LSEC cell line TSEC [15] was provided by Dr. Vijay Shah (Mayo Clinic, Rochester, MN) and was cultured in Dulbecco’s modified Eagle medium (DMEM) with 10% fetal bovine serum (FBS), 1% Pen/Strep (Invitrogen) and 1% Endothelial Cell Growth Supplement (Sciencell). Human immortalized LSEC (TMNK-1) [16] were kindly provided by Dr. Alejandro Soto-Gutierrez (University of Pittsburgh, PA) and were cultured in DMEM supplemented with 10% FBS and 1% penicillin/streptomycin (Invitrogen). The murine (AML12) and human (HepG2) hepatocyte cell lines were cultured according to the vendors instructions (ATCC, Manassas, VA).

Eight-week-old male C57Bl/6 mice and sixteen-week-old male mice fed a 60% fat diet (380050 DIO) or a 10% fat diet (380056 NCD) for 12 weeks were purchased from Jackson Laboratories (Bar Harbor, ME). All animal experiments were performed humanely as approved by the Animal Care and Use Committee at the University of Colorado Denver under protocol number B-94114(02)1D. Animals were sacrificed using CO2 asphyxiation followed by cervical dislocation and the appropriate organs were harvested following sacrifice.

### Cell Culture

AML12 and TSEC cell lines (5x10^5^ and 1.25x10^5^ respectively) were plated in 24 well plates with culture media (mentioned previously). After cells had adhered, media was replaced with media containing 1% FFA-free bovine serum albumin (BSA) (Sigma) and variable concentrations of the long chain FFAs palmitic acid (PA, Sigma), oleic acid (OA, Sigma) or both. 100mM stock solutions of PA and OA were made up in DMSO and fatty acid were pre-incubated with 2.5mM FFA-free BSA prior to adding to culture. For LPS stimulation 100ng/ml LPS (Sigma) was added to the wells for 6 hours. For signaling experiments the MEK1/2 inhibitor PD-0325901 (EMD Calbiochem) was added to LSEC culture 1 hour prior to treatment with FFA. Stock solutions of PD-0325901 were made up at 100mM in DMSO and 1 and 0.3**μ**M were added to the wells. RNA was isolated for quantitative PCR and CCL2 levels in the supernatants were determined using the mouse CCL2 DuoSet ELISA according to the standard protocol (R&D).

### Microarray Analysis

For microarray analysis triplicate wells of the murine LSEC line, TSEC, were treated with PA and OA (0.33mM/0.33mM) for 18 hours. RNA was harvested using RNeasy Mini kit (Qiagen) and RNA quality verified using the Eukaryote Total RNA Nano assay on an Agilent 2100 Bioanalyzer (Agilent Technologies). Gene expression profiling was performed using Mouse GeneChip ST 2.0 arrays (Affymetrix). Partek® Genomics Suite™ ver.6.6 (www.partek.com) was used to process and perform the statistical analysis on these array data, using GC-RMA to compute log2 expression values. A gene was considered present if it had an expression value > 4.5 in at least one sample (26,084 out of 41,353 transcripts total). A P-value <0.05 (ANOVA) was used to define differential expression in all present genes (765 out of 26,084 transcripts). The enrichment of KEGG pathways among significantly regulated genes was determined using WebGestalt (http://bioinfo.vanderbilt.edu/webgestalt). Specific pathways and upstream regulators altered by FFA treatment were determined using Ingenuity Pathway Analysis (IPA) software (Qiagen). Microarray data is available in the Gene Expression Omnibus (GEO) repository (NCBI) under the accession # GSE67651.

### Flow Cytometric Analysis

Multiparameter flow cytometry was performed using a BD FACSCanto II instrument (BD Biosciences) analyzed using FACSDiva software (BD Biosciences). Fluorochrome-labeled monoclonal antibodies specific for mouse CD45 (30-F11), CD11b (M1/70), F480 (BM8), Ly6G (RB6-8C5) and Ly6C (HK1.4) were obtained from Affymetrix. Anti-mouse CD146 (Clone ME-9F1) was from BD Biosciences and anti-mouse Stabilin-2 (Clone 34–2) was from MBL International. Cells were stained in phosphate-buffered saline (PBS) containing 1% bovine serum albumin and 0.01% sodium azide at 4°C in the dark for 45 min, washed twice and subsequently fixed in 200 **μ**l of stabilizing fixative (BD). Isotype-matched control antibodies were used to determine background levels of staining.

### Isolation of Liver Sinusoidal Endothelial Cells

For LSEC isolation mouse livers were perfused with 40ml perfusion buffer (10mM Hepes-NaOH, pH 7.4, 150mM NaCl, 5mM KCl, 1mM MgCl2) containing 30ug/ml liberase (Roche) prior to removal followed by homogenization with a 10cc syringe back. Red blood cells were lysed in lysis buffer (0.16M NH_4_Cl, 0.17M TRIS (pH 7.65)) and the cells were washed twice in RPMI 1640 (Gibco) with 5% fetal bovine serum (HyClone). Cells were filtered through 100um filter followed by density gradient separation with 20% OptiPrep (Sigma). Cells were further purified using magnetic bead separation columns (Miltinyi) where CD45+ cells were depleted followed by positive selection of CD146^+^ cells [17]. DiI-AcLDL uptake was determined by incubation with 10ug/ml DiI-conjugated Ac-LDL (Life Technologies) for 4 h at 5% CO2 at 37°C followed by flow cytometric analysis of DiI-AcLDL uptake.

### Scanning Electron Microscopy

Freshly isolated LSEC from mice were plated at 1 x 10^6^/ml on collagen-coated cover slips in a 24 well plate and incubated at 37C for 8 hours. Cells were fixed in 2.5% glutaraldehyde for 2 hours. Following a cacodylate buffer rinse, the cells were secondary fixed in 1% OsO4, then rinsed again in buffer and dehydrated in EtOH solutions, 50%, 70%, 90%, 100% (X2), for 15min each. Following dehydration, the cells were critical point dried in a LEICA EM CPD 300 using 100% EtOH as the transition liquid. The coverslips were mounted on SEM stubs with double sided carbon tape and sputter coated with gold/palladium for 30 seconds using the Leica EM ACE200. The samples were then imaged using a JEOL JSM-6010LA SEM operating at 10kV.

### Annexin V Staining

TSEC were plated at 1.25x10^5^ and incubated for 18 hours with varying concentrations of FFA. Staining was performed using the FITC Annexin V Apoptosis Detection Kit I (BD Biosciences) and the percentage of apoptotic (Annexin V+) cells was evaluated by flow cytometric analysis.

### Quantitative PCR

RNA from was extracted using the RNeasy Mini Kit and converted to cDNA using the QuantiTect RT kit (both from Qiagen, standard protocols). Real Time Quantitative PCR was carried out on a 7300 Real Time PCR system (Applied Biosystems). The following Quantitect primer pairs were purchased from Qiagen; 18s, CCL2, CCL7, CXCL10, CXCL12, CXCL16. Samples were run in triplicate in a 10ul reaction volume consisting of 5ul of SYBR Green PCR Mix (Qiagen), 1ul of the primer set, 0.5ul of cDNA and 3.5ul of H_2_O. Cycling conditions consisted of 40 cycles (94c for 15 seconds, 5 0c for 30 seconds, 72c for 30 seconds). Each individual sample was normalized to 18s and gene expression was compared to the matched untreated sample. Fold change in transcripts was calculated using the ΔΔCT method [18].

### Cellular Migration Assay

For the cellular migration assays tissue culture-treated 6.5 mm transwells with 5.0 **μ**m pore polycarbonate membrane inserts were placed in 24 well plates containing 1.25x10^6^ TSEC or 5x10^6^ AML12 cells either untreated or incubated with LPS (100ng/ml) and FFA (0.33uM PA acid and 0.33uM OA) and equilibrated for 1 hour. Bone marrow monocytes were isolated by negative bead selection from C57BL6 bone marrow using a monocyte isolation kit (Miltenyi). 0.5–1 x10^6^ monocytes were added to the transwell in 100ul volume and incubated at 37C for 3 hours. Following incubation transwells were removed and the wells were harvested and stained with fluorescently labeled antibodies against CD45, CD11b, F480, Ly6G and Ly6C. 5x10^4^ polystyrene/divinylbenzene beads (Bang Laboratories PS07N/9400) were added to the tube and 10,000 beads per sample were collected by flow cytometry. The total number of migrated cells was calculated by dividing the number of counted cells by the number of counted beads and multiplying this number by 50,000 (the total number of beads in the sample) [(# of counted cells/# of counted beads) x 50,000]. The chemotactic index was determined by dividing the number of migrated beads in the experimental wells by the number of migrated beads in the control wells.

### Statistical Analysis

Results were expressed as the mean +/- SEM. Unpaired Student’s t tests were used to compare differences between groups and a p value of ≤0.05 was considered significant. The Prizm 5.0 statistical analysis software was used (GraphPad Software).

## Results

### FFA Increase Lipid Metabolism and Signaling Pathways in a Murine LSEC Cell Line

**Fig 1:**
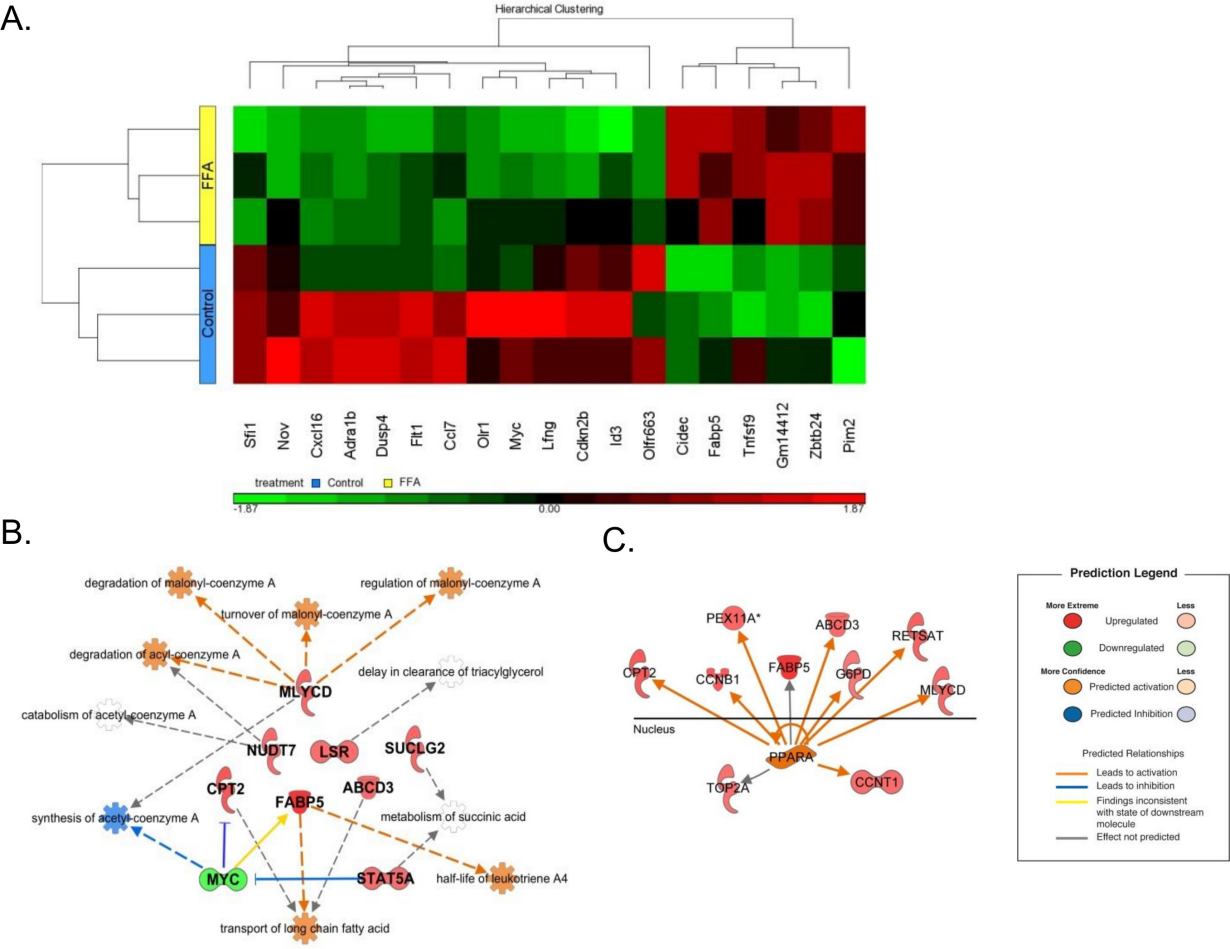
Gene expression analysis of TSEC treated with FFA. TSECs were treated with 0.33mM OA and 0.33mM PA for 16 hours. RNA was isolated from cells and microarray analysis of triplicate wells was performed using IPA (Qiagen). (A) Heat map of the top 20 up-regulated and down-regulated genes in the FFA treated LSEC compared to untreated cells. Individual samples are given on the rows, while genes are listed by column. Gene expression is normalized to the mean and color-scaled by standard deviations above (red) and below (green) the average expression in all six independent samples. (B) Upregulation of genes involved in lipid metabolism genes (red) and the predicted activated pathways (orange) in TSEC treated with FFA. (C) Predicted activation of PPAR**α** based on Ingenuity upstream regulator analysis. Upregulated genes downstream of PPAR**α** are shown (red).

**Fig 2:**
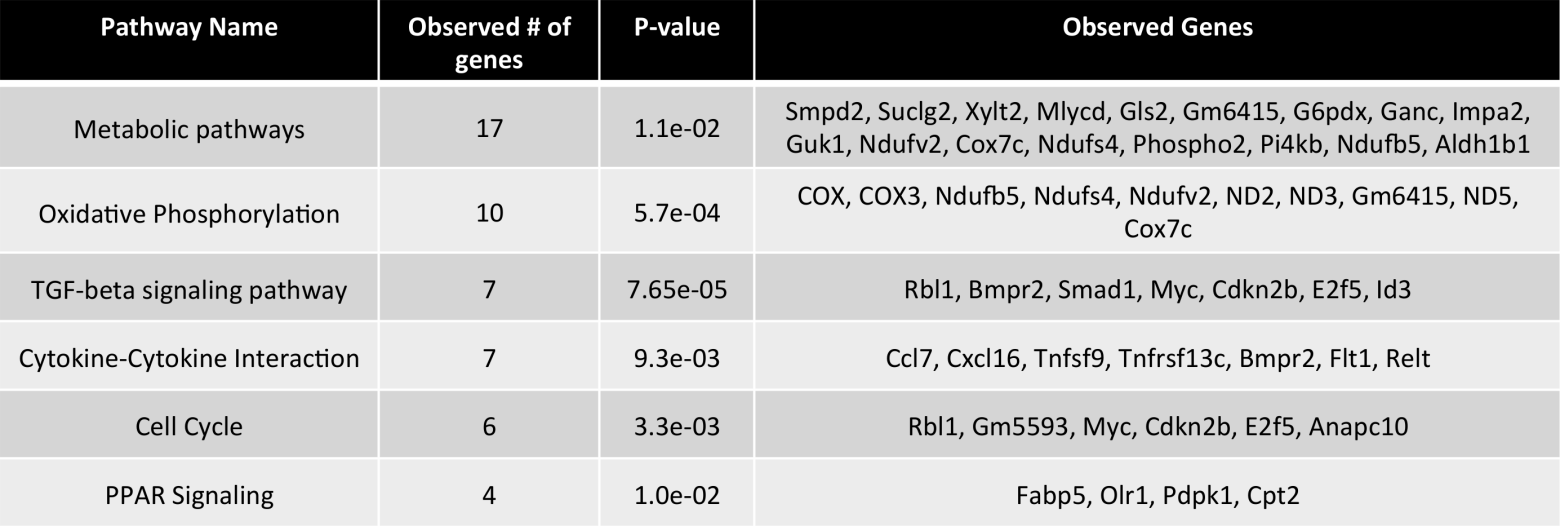
KEGG pathways in FFA treated TSEC. Relevant enriched pathways and the involved genes were determined by analysis of all genes with a p value <0.05 and a fold-change > 1.25 within the KEGG pathway database.

PA (C16:0) and OA (C18:1) are the most abundant FFA in normal and NAFLD livers, and treatment of hepatocytes with these FFA has been shown to mimic fat over- accumulation in the liver seen in NAFLD [19,20]. To begin to determine the effects of free fatty acids on LSEC, we treated the murine LSEC line (TSEC) with OA and PA (0.33mM/0.33mM) for 18 hours. This assay has been shown to produce significant steatosis in hepatocytes with minimal induction of apoptosis [19]. We also observed minimal induction of apoptosis in LSEC at the concentration of FFA used (S1 Fig). We assayed global transcriptional changes in TSEC treated with FFA using the Mouse GeneChip ST 2.0 (www.affymetrix.com). Treatment with FFA led to a significant change in 765 gene transcripts (p<0.05, ANOVA) when compared to untreated cells (full microarray data is available in the Gene Expression Omnibus (GEO) repository (NCBI) under the accession # GSE67651). The top 20 up- and down-regulated genes are shown in Fig 1A and the relevant KEGG pathways enriched in FFA treated TSEC was determined using WebGestalt (Fig 2). These included metabolic pathways, oxidative phosphorylation pathways and PPAR signaling pathways. Similar pathways were also found to be activated using Ingenuity pathway analysis with many of the upregulated genes being important in lipid metabolism (Fig 1B). Genes downstream of the fatty acid nuclear receptor PPAR**α** were also significantly increased, suggesting activation of PPAR**α** (evidence for activation > 2 Std. dev. from random noise) (Fig 1C). Overall, this data suggests that the LSEC line demonstrates active lipid metabolism pathways.

### FFA Inhibit Chemokine Production from Murine and Human LSEC Cell Lines

**Fig 3:**
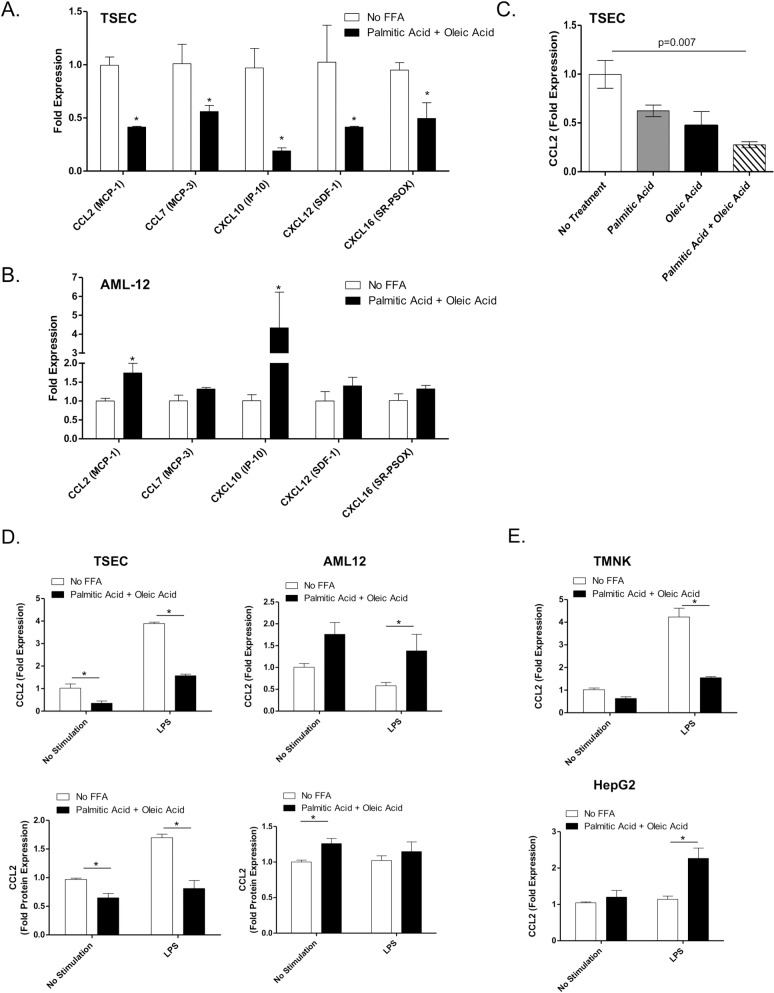
Downregulation of chemokines by LSEC in response to treatment with FFA. TSEC (A) and AML12 (B) were cultured with 0.33mM OA and/or 0.33mM PA for 16 hours. RNA was isolated from cells and levels of the indicated chemokine were determined by quantitative RT PCR. (C) TSEC were treated with 0.33mM of the indicated FFA for 16 hours and CCL2 expression was determined by quantitative RT PCR. (D) TSEC and AML12 were treated with 0.33mM OA and 0.33mM PA for 16 hours followed by 6 hour stimulation with 100ng/ml LPS. CCL2 gene expression (top) and protein production (bottom) were measured. (E) The human LSEC (TMNK) and hepatocyte (HepG2) cell lines were stimulated with FFA and LPS as in (D) and CCL2 gene expression was measured by quantitative PCR. Plots represent the mean +/- SE of three experiments. *p<0.05.

In addition activation of metabolic pathways, genes involved cytokine-cytokine interaction pathways were also found to be enhanced (Fig 2). Interestingly, two of the top downregulated genes following FFA treatment were chemokines (CCL7 and CXCL16; Fig 1A). CCL7 has been shown to be important for the recruitment of Ly6C^high^ pro-inflammatory monocytes to inflamed tissues [21], whereas CXCL16 is a chemoattractant for NKT cells [22]. Based on the observed downregulation of CCL7 and CXCL16 by TSEC following treatment with FFA, we decided to evaluate the transcriptional expression of a panel of chemokines following treatment to determine if other chemokines were similarly downregulated. TSECs were treated with PA and OA for 16 hours and chemokine gene expression was analyzed (Fig 3A). In agreement with the microarray data, we observed FFA-mediated transcriptional inhibition of both CCL7 and CXCL16. We also observed a similar downregulation of other chemokines involved in monocyte and macrophage recruitment in response to treatment including CCL2, CXCL10 and CXCL12. A decrease in chemokine gene expression was not observed in the mouse hepatocyte cell line AML12 when treated with FFA (Fig 3B), indicating this response may be specific to LSECs. Individually, both PA and OA had mild inhibitory effects on CCL2 production but the effect was most pronounced when cells were treated with both PA and OA together (Fig 3C).

As the pathogenesis of NAFLD has been clearly linked with the recruitment of pro-inflammatory monocytes to the liver, we focused on CCL2 whose genetic absence has been shown to abrogate NAFLD [23]. FFA not only downregulated CCL2 in resting TSEC but significantly inhibited LPS-induced CCL2 gene expression and protein production in both murine (TSEC) and human (TMNK) LSEC lines (Fig 3D and 3E). This was in contrast to murine (AML12) and human (HepG2) hepatocyte cell lines which were sensitized to LPS-induced CCL2 production by FFA (Fig 3D and 3E).

### FFA Alter TSEC Chemokine Expression through a MAPK Dependent Pathway

FFA can act as ligands for and activate a number of cell surface and nuclear receptors. We therefore evaluated the effects of inhibition of GPR40, PPAR**α**, PPAR**γ** or PPAR**δ** pathways with small molecule inhibitors on the effects of FFA. We did not see any effect of inhibition of these receptors on chemokine regulation by treatment with FFA (data not shown). However, when MAPK signaling was inhibited using the MEK1/2 inhibitor PD-0325901 the FAA-induced chemokine downregulation was reduced in a dose dependent manner (S2 Fig).

### FFA Inhibit TSEC-Mediated Monocyte Migration

**Fig 4:**
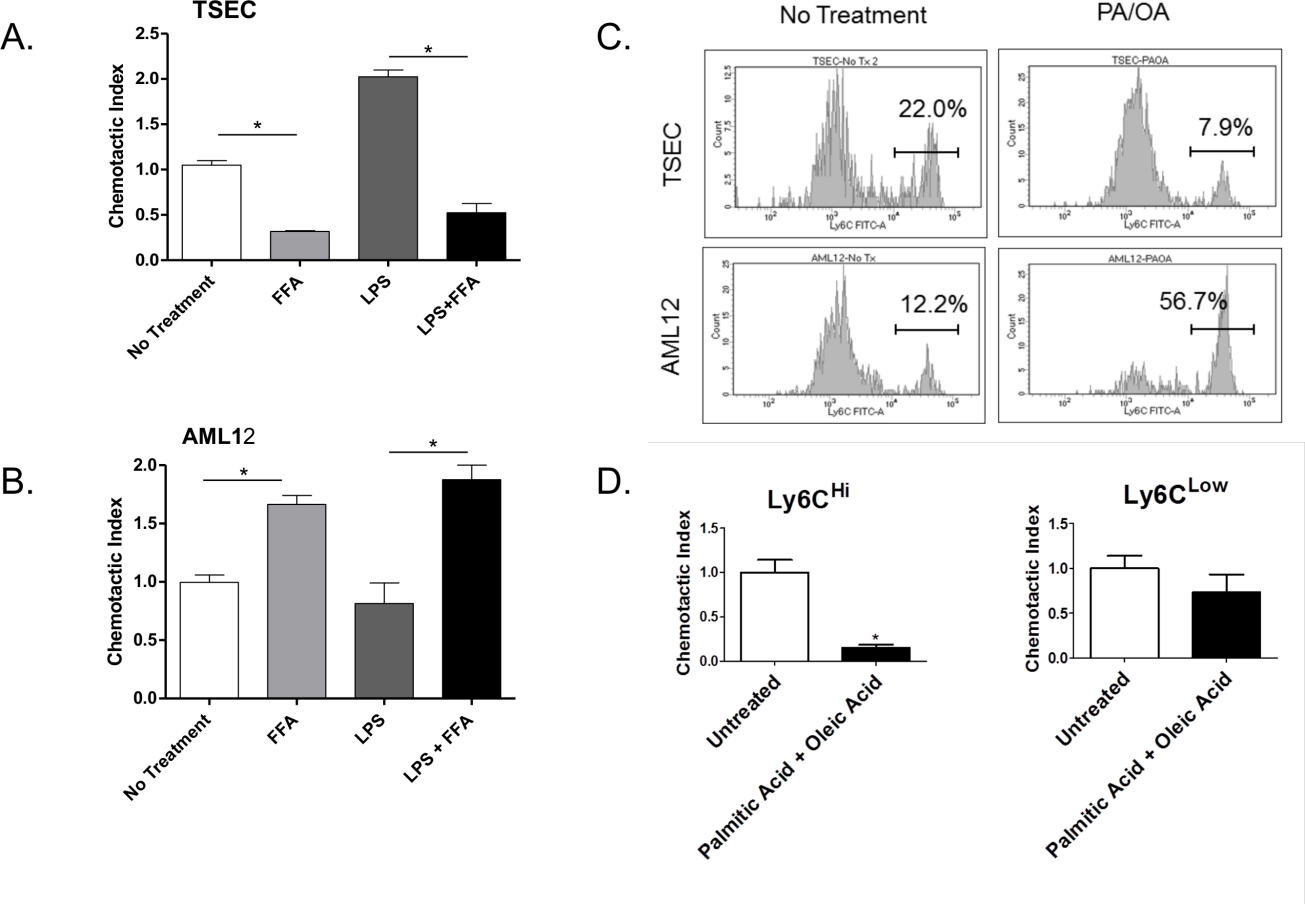
FFA inhibits the migration of monocytes in vitro. Murine monocytes were isolated from the bone marrow of C56Bl6/J mice and co-cultured for 2 hours in transwell plates with TSEC (A) or AML12 (B) either resting or pre-treated with FFA and LPS for 18 hours. Cells were stained with the monocyte/macrophage markers CD11b, F/480 and Ly6C and the chemotactic index was calculated (#migrated cells in treatment wells/# migrated cell in the control well). (C) Representative histograms showing Ly6C expression on migrated cells. (D) The chemotactic index of Ly6C high and Ly6C low cells towards resting or FFA-treated TSEC. Plots represent the mean +/- SE of three experiments. *p<0.05.

Since CCL2 has been shown to be involved in the recruitment of monocytes to the liver we evaluated the effects of FFA treatment on the recruitment of monocytes by TSEC using an *in vitro* migration assay. Following treatment of the LSEC cell line with FFA we saw a decrease in the chemotactic response of CD11b^+^Ly6G^-^ monocytes to both resting and LPS stimulated TSEC (Fig 4A). In agreement with the CCL2 expression data in Fig 3, FFA did not inhibit hepatocyte-induced monocyte recruitment but enhanced LPS-induced migration of monocytes (Fig 4B). Analysis of the phenotype of the monocytes demonstrated lower expression of Ly6C on the migrated cells (Fig 4C and 4D) suggesting that the FFA inhibit the recruitment of Ly6C^high^ cells. Ly6C^high^ monocytes are associated with a pro-inflammatory phenotype in both obesity and NALFD [24]. These results demonstrate that the TSEC recruit monocytes in response to LPS stimulation and that high FFA levels may inhibit the recruitment of pro-inflammatory monocytes by these cells.

### Expression of Chemokines in LSEC Isolated from Lean and Obese Mice

We have shown that FFA can alter chemokine expression in the LSEC cell lines. The murine TSEC line has been shown retain an endothelial cell genetic signature in addition to displaying a number of LSEC-specific functions including VEGF and FGF induced migration, AcLDL uptake and secretion of matrix remodeling proteins [15]. However, as with all cell lines, there is the possibility of functional and phenotypical divergence from primary cells as a result of passage in culture, therefore, we next evaluated the effects of FFA on primary LSECs.

**Fig 5:**
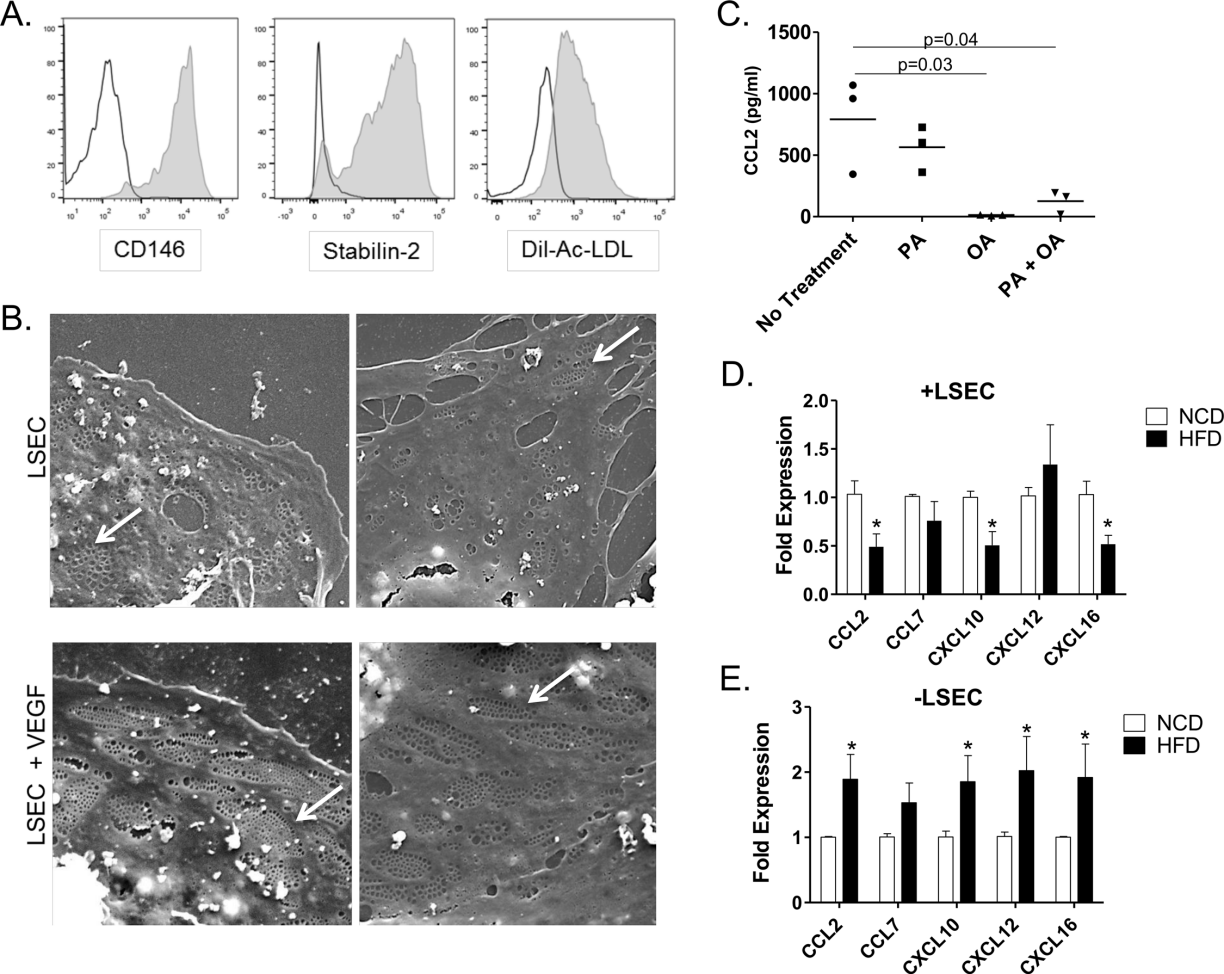
Phenotype of LSEC from normal and DIO mice. A) Primary LSEC (CD45^-^CD146^+^) isolated from normal C57Bl6 lean mice were analyzed for CD146 and CD31 expression and uptake of DiI-labeled Ac-LDL by flow cytometry. (B) Primary LSEC isolated from C57Bl6 lean mice were cultured with the indicated FFA for 24hours. CCL2 levels in the supernatant were measured by ELISA. Graphs represent the mean +/- SE from 3 mice. Primary LSEC (CD45^-^CD146^+^) (C) and LSEC depleted non-parenchymal cells (CD45^+^CD146^-^) (D) were isolated from mice fed a low fat diet (NCD) and obese mice fed a high fat diet (HFD) for 12 weeks and gene expression for chemokines was evaluated by quantitative qPCR. Graphs represent the mean +/- SE from 5–7 mice. *p<0.05.

Primary LSEC (defined as CD45^-^CD146^+^) were isolated from the livers of chow-fed C67Bl/6 mice by magnetic bead separation. These cells were greater than 98% positive for both CD146 and Stabilin-2, markers of LSEC in mice [11,17](Fig 5A). To further determine the purity of primary LSECs we performed an acetylated LDL (AcLDL) uptake assay [17]. Following incubation with DiL-conjugated AcLDL for 4 hours the isolated LSEC maintained the ability to endocytose the labeled AcLDL as measured by flow cytometry (Fig 5A). Finally, the presence of fenestrations, a hallmark of LSEC phenotype, was verified using scanning electron microscopy (Fig 5B). Primary LSECs isolated from mice constitutively produced CCL2 protein in culture and, as seen with the TSEC line, addition of FFA directly inhibited its production (Fig 5C). In primary cells this downregulation appears to result primarily from OA since PA did not significantly downregulate CCL2.

Finally, we determined whether LSEC displayed an altered phenotype in the context of in vivo NAFLD where increased FFA are likely encountered by these cells in the steatotic liver. We isolated LSEC from obese DIO mice fed a normal control low-fat diet (NCD) or a high fat diet (HFD) for 12 weeks. Gene expression analysis of LSEC isolated from obese mice show a significantly lower expression of CCL2, CXCL10 and CXCL16 in CD45^-^CD146^+^ LSEC when compared to those from mice fed a NCD (Fig 5D). The downregulation of these chemokines is in agreement with the in vitro FFA treatment data. CCL7 and CXCL12 were not decreased in LSEC from a steatotic liver suggesting some differences between the cell line and in vivo LSEC environment. However, non-parenchymal cells (CD45^+^CD146^-^) from steatotic livers demonstrated expressed significantly more CCL2, CXCL10, CXCL12 and CXCL16 than those from a normal liver (Fig 5E). Thus, the ex vivo data indicates that a high-fat diet induces different alterations in the transcriptional expression of pro-inflammatory chemokines within the population of LSEC compared to other hepatic non-parenchymal cells.

## Discussion

LSEC have been shown to have both pro- and anti-inflammatory functions in the liver [13,25]. While some endothelial cell dysfunction has been demonstrated in animal models of NAFLD [26,27] the contribution of LSEC in the inflammatory response in NAFLD has not been fully elucidated. NAFLD is characterized by increased hepatic accumulation of lipids, likely resulting from multiple factors including lipolysis from adipocytes or increased intake of dietary fat followed by enhanced FFA production, as well as de novo lipogenesis and impaired lipid disposal [3,28]. Increased exposure of hepatocytes and macrophages to FFA can lead to lipotoxicity, increased pro-inflammatory cytokine production and liver injury [20,29]. Therefore, in the current study, we investigated the effects of increased FFA on another predominant hepatic cell type, LSEC, which are important for transport of lipids and lipoproteins across the sinusoids. Here, we show that culture of LSECs with free fatty acids leads to an upregulation of lipid metabolism pathways and a down-regulation of pro-inflammatory chemokines. To our knowledge, this is the first study demonstrating that LSECs exposed to FFA develop an anti-inflammatory profile, including decreased chemokine gene and protein expression and reduced recruitment of immune cells.

Chemokine expression on and by endothelial cells is important for immune cell recruitment, adhesion and transmigration through the endothelium [30,31]. In the current study, we found a significant FFA-induced downregulation of a number of chemokines including the CC chemokines CCL2 (MCP-1) and CCL7 (MCP-3) and the CXC chemokines CXCL10 (IP-10), CXCL12 (SDF-1) and CXCL16 (SR-PSOX). CCL2 has been shown to be particularly relevant to NAFLD where it’s involved in recruitment of inflammatory cells expressing its receptor CCR2. CCR2 is expressed primarily on monocytes and macrophages but can also be found of subsets of lymphocytes and NK cells [32,33]. In addition, CCL2 has also been shown to be increased in livers of mice with high-fat diet induced steatosis [34]. While we initially hypothesized that FFA would enhance the pro-inflammatory response of LSEC we instead saw a consistent decrease suggesting that cells LSEC are not responsible for the increased recruitment of these cells in the context of high FFA levels in the liver. Interestingly, we observed differing effects of FFA on chemokine expression in hepatocytes when compared to LSEC. While FFAs appear to inhibit LSEC-produced chemokines, they have no effect or sensitize hepatocytes to LPS-induced chemokine production suggesting that *in vivo* LSECs help to compensate for the inflammation induced by increased FFA in the liver. This would be in agreement with studies demonstrating an anti-inflammatory role for LSEC in alcoholic liver disease [25]. Similarly, LSEC have been shown to inhibit stellate cell activation [35], thus attenuating fibrosis development.

The murine TSEC cell line used in these experiments has been shown to retain a number of LSEC- specific functions including endocytosis of AcLDL, vascular tube formation on Matrigel and migration in response to angiogenic growth factors [15]. However, these cells have a limited number of fenestrae organized in sieve plates and display an “activated” angiogenic phenotype suggesting they may not represent a “resting” phenotype. Therefore, we investigated chemokine expression in primary LSEC (CD45^-^CD146^+^) isolated from normal non-obese mice and mice fed a high fat diet. We found that FFAs were also able to inhibit CCL2 production from primary LSEC, verifying that the effect was not specific to the cell line. LSEC from steatotic livers also displayed significantly lower expression of CCL2, CXCL10 and CXCL16 in agreement with the in vitro cell line data. CCL2, CXCL10 and CXCL16 have all been shown to be expressed in LSEC and can recruit monocytes, T cells, NK cells and NKT cells in acute liver injury [33,36]. CCL7 and CXCL12 were neither significantly upregulated nor downregulated in LSEC from steatotic livers which was different from what was observed in the cell lines and likely reflects the complexity of the factors altering LSEC phenotype in the in vivo environment. Overall in the steatotic liver LSEC appear to response to increased FFA in a manner different from other hepatic cells since hepatic lymphocytes and monocytes (i.e., CD45^+^CD146^-^) isolated from steatotic livers had higher expression of a number of pro-inflammatory chemokines.

FFA can act as ligands for and activate a number of cell surface and nuclear receptors. The function of oleic acid has been shown to be at least in part due to GPR40 activation in certain cell lines [37]. In addition, FFA can act as ligands for peroxisome proliferator-activated receptors which have been shown to have anti-inflammatory effects via NFkB inhibition [38,39]. It is plausible that activation of one of these pathways could be responsible for the observed effects of FFA on chemokine expression seen here. However, in our studies, we did not see any effect of inhibition of GPR40, PPAR**α**, PPAR**γ** or PPAR**δ** pathways with small molecule inhibitors on chemokine regulation by treatment with FFA (data not shown). We were able to demonstrate that the anti-inflammatory effects of FFA in LSEC are dependent on MAPK signaling since inhibition of MEK reversed the effect. Interestingly lipid-induced MAPK signaling has been shown to be important for cell survival in LSEC [40]. The possibility remains that there are other free fatty acid-binding surface or nuclear receptors that respond to FFA and inhibit chemokine production, and future studies will be performed to further clarify the mechanism.

Taken together, our data points to the liver’s remarkably diverse inflammatory responses to stimuli, in this case FFA. Since NASH is characterized by increased infiltration of pro-inflammatory cells, in the context of increased hepatic uptake of FFA, LSECs may provide a compensatory mechanism whereby they downregulate chemokines and help prevent disease progression. Further studies aimed at understanding how LSECs might protect against liver injury in NALFD are warranted.

## Supporting Information

S1 Fig Free fatty acids do not induce apoptosis of the TSEC line. (PDF)

S2 Fig Downregulation of chemokines by FFAs is dependent on MAPK signaling. (PDF)
