# Correction: Free Fatty Acids Differentially Downregulate Chemokines in Liver Sinusoidal Endothelial Cells: Insights into Non-Alcoholic Fatty Liver Disease.

**DOI:** 10.1371/journal.pone.0168301

**Published:** 2016-12-08

**Authors:** Rachel H. McMahan, Cara E. Porsche, Michael G. Edwards, Hugo R. Rosen

There are a number of errors in the caption for Fig 5. Please see the complete, correct Fig 5 caption here:

**Fig 5:**
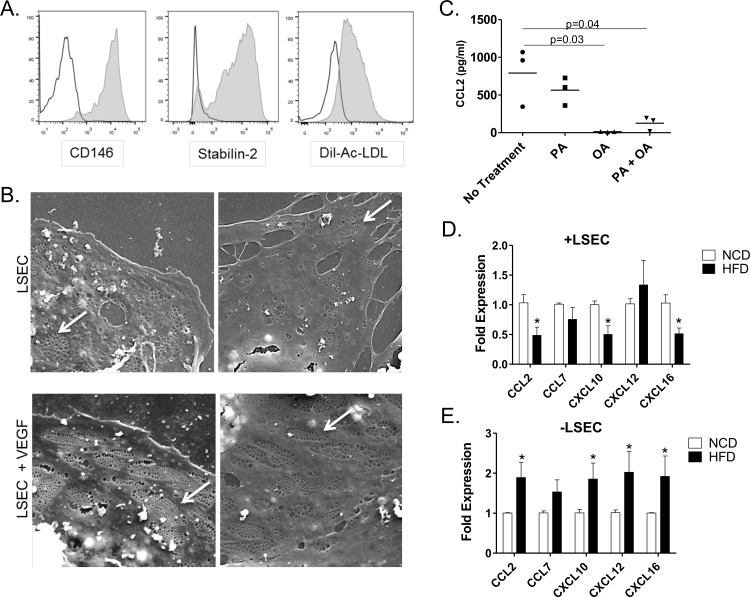
Phenotype of LSEC from normal and DIO mice. (A) Primary LSEC (CD45^-^CD146^+^) isolated from normal C57Bl6 lean mice were analyzed for CD146 and Stabilin-2 expression and uptake of DiI-labeled Ac-LDL by flow cytometry. (B) Scanning electron microscopy of fenestrae (arrows) in LSEC directly isolated from C57Bl6 mice cultured with and without 10ng/ml VEGF (5000x). (C) Primary LSEC isolated from C57Bl6 lean mice were cultured with the indicated FFA for 24hours. CCL2 levels in the supernatant were measured by ELISA. Graphs represent the mean +/- SE from 3 mice. Primary LSEC (CD45^-^CD146^+^) (D) and LSEC depleted non-parenchymal cells (CD45^+^CD146^-^) (E) were isolated from mice fed a low fat diet (NCD) and obese mice fed a high fat diet (HFD) for 12 weeks and gene expression for chemokines was evaluated by quantitative qPCR. Graphs represent the mean +/- SE from 5–7 mice. *p<0.05
