# Supplementary material for: Free Fatty Acids Differentially Downregulate Chemokines in Liver Sinusoidal Endothelial Cells: Insights into Non-Alcoholic Fatty Liver Disease
Source: PLoS One. 2016 Jul 25;11(7):e0159217. doi: 10.1371/journal.pone.0159217 (PMC4959750; doi:10.1371/journal.pone.0159217)
Supplement: S1 Fig — (PDF) [file pone.0159217.s001.pdf]

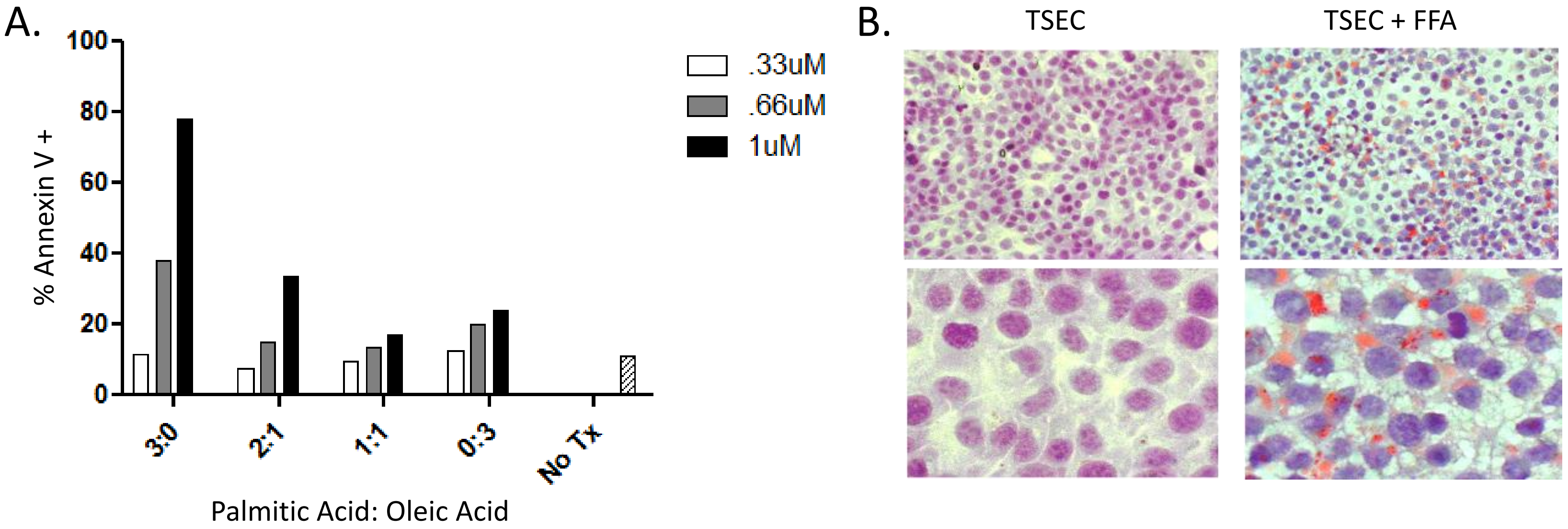


**S1 Fig. Free fatty acids do not induce apoptosis of the TSEC line.** Murine LSEC cells (TSECs) were treated with the indicated ratio and concentration of FFA for 18 hours. (A) Apoptosis was assessed by flow cytometric analysis of Annexin V staining. (B) Lipid accumulation in cells treated with 0.33mM PA/0.33mM PA was assayed by oil red O staining.
