# Supplementary material for: Free Fatty Acids Differentially Downregulate Chemokines in Liver Sinusoidal Endothelial Cells: Insights into Non-Alcoholic Fatty Liver Disease
Source: PLoS One. 2016 Jul 25;11(7):e0159217. doi: 10.1371/journal.pone.0159217 (PMC4959750; doi:10.1371/journal.pone.0159217)
Supplement: S2 Fig — (PDF) [file pone.0159217.s002.pdf]

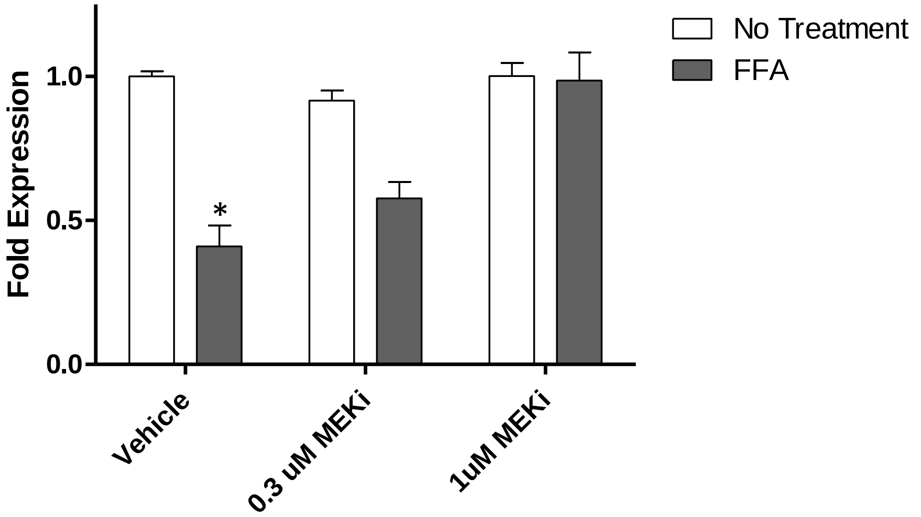

S2 Fig. Downregulation of chemokines by FFAs is dependent on MAPK signaling. TSEC were treated with the indicated concentration of the MEK1/2 inhibitor (MEKi) for 1 hour. Cultures were then incubated with FFA for 18 hours and CCL2 gene expression was measured by quantitative PCR. Plots represent the mean +/- SE of triplicate experiments. *p<0.05
